# Natural Bizbenzoquinoline Derivatives Protect Zebrafish Lateral Line Sensory Hair Cells from Aminoglycoside Toxicity

**DOI:** 10.3389/fncel.2016.00083

**Published:** 2016-03-30

**Authors:** Matthew Kruger, Robert Boney, Alexander J. Ordoobadi, Thomas F. Sommers, Josef G. Trapani, Allison B. Coffin

**Affiliations:** ^1^School of Biological Sciences, Washington State UniversityVancouver, WA, USA; ^2^College of Arts and Sciences, Washington State UniversityVancouver, WA, USA; ^3^Department of Biology and Neuroscience Program, Amherst CollegeAmherst, MA, USA; ^4^Department of Integrative Physiology and Neuroscience, Washington State UniversityVancouver, WA, USA

**Keywords:** zebrafish, hair cell protection, hearing loss, aminoglycosides, lateral line, ototoxicity, cisplatin

## Abstract

Moderate to severe hearing loss affects 360 million people worldwide and most often results from damage to sensory hair cells. Hair cell damage can result from aging, genetic mutations, excess noise exposure, and certain medications including aminoglycoside antibiotics. Aminoglycosides are effective at treating infections associated with cystic fibrosis and other life-threatening conditions such as sepsis, but cause hearing loss in 20–30% of patients. It is therefore imperative to develop new therapies to combat hearing loss and allow safe use of these potent antibiotics. We approach this drug discovery question using the larval zebrafish lateral line because zebrafish hair cells are structurally and functionally similar to mammalian inner ear hair cells and respond similarly to toxins. We screened a library of 502 natural compounds in order to identify novel hair cell protectants. Our screen identified four bisbenzylisoquinoline derivatives: berbamine, E6 berbamine, hernandezine, and isotetrandrine, each of which robustly protected hair cells from aminoglycoside-induced damage. Using fluorescence microscopy and electrophysiology, we demonstrated that the natural compounds confer protection by reducing antibiotic uptake into hair cells and showed that hair cells remain functional during and after incubation in E6 berbamine. We also determined that these natural compounds do not reduce antibiotic efficacy. Together, these natural compounds represent a novel source of possible otoprotective drugs that may offer therapeutic options for patients receiving aminoglycoside treatment.

## Introduction

Over 360 million people worldwide are affected by moderate to severe hearing impairment, including 37.5 million Americans (Blackwell et al., 2014; World Health Organization, 2015). Hearing loss can have devastating consequences including social isolation, decreased employment opportunities, and a loss of household income (Kochkin, 2007; Mick et al., 2014; Emmett and Francis, 2015). Hearing loss is caused by genetic mutations, aging, loud noise, and exposure to certain ototoxic medications such as the aminoglycoside antibiotics neomycin or gentamicin (Fee, 1980; Lenz and Avraham, 2011; Schacht et al., 2012; Furness, 2015). Aminoglycosides are commonly prescribed to treat antibiotic resistant gram-negative bacterial infections such as tuberculosis (Durante-Mangoni et al., 2009), neonatal infections, and infections associated with cystic fibrosis (Rizzi and Hirose, 2007). For example, in cystic fibrosis patients, infection caused by *Pseudomonas aeruginosa* responds positively to aminoglycoside treatment (Vázquez-Espinosa et al., 2015). However, as a side effect of treatment, approximately 20–30% of patients suffer from ototoxic damage (Rizzi and Hirose, 2007; Xie et al., 2011; Schacht et al., 2012). Methods are needed to ameliorate this damage and promote safe use of these antibiotics.

Aminoglycoside-induced hearing loss results from damage to sensory hair cells of the inner ear (Schacht et al., 2012). Aminoglycosides kill hair cells via activation of multiple signaling cascades, including programmed cell death pathways (Forge and Schacht, 2000; Matsui et al., 2002; Jiang et al., 2006; Coffin et al., 2013b). Aminoglycoside exposure is correlated with increased reactive oxygen species, a loss of mitochondrial membrane potential, and subsequent hair cell death, sometimes accompanied by signs of classical apoptosis such as nuclear condensation and caspase activation (Forge and Li, 2000; Matsui et al., 2002, 2004; Hirose et al., 2004; Owens et al., 2007). However, several lines of evidence suggest that different aminoglycosides may activate different cell death pathways and that even a single aminoglycoside may act on multiple signaling pathways within a single sensory epithelium (Jiang et al., 2006; Owens et al., 2009; Coffin et al., 2013a,b). For example, Jiang et al. (2006) found variable cell morphology in cochleae from aminoglycoside-treated mice, indicative of multiple modes of cell death. Furthermore, they did not find evidence for caspase activation, but rather for activation of other proteases such as calpains and cathepsins. Similarly, aminoglycoside toxicity in the zebrafish lateral line is likely caspase-independent (Coffin et al., 2013b). Different aminoglycosides also activate only partially-overlapping cell death pathways in the lateral line, with neomycin activating mitochondrially-associated signaling via Bax, and gentamicin activating Bax-independent mechanisms that act through p53 (Owens et al., 2009; Coffin et al., 2013a). Compounds that modulate these intracellular signaling pathways offer therapeutic options for preventing aminoglycoside ototoxicity. However, given the complexity of the cell signaling events involved, it is often difficult to take an *a prioi* approach to selecting a single molecular target for manipulation. We have therefore adopted an objective screen with the goal of identifying one or more natural compounds that prevent aminoglycoside ototoxicity.

Natural compounds such as plant extracts offer a novel source of otoprotective drugs. Natural compounds have been used in Eastern medicine for thousands of years and are still used today by people around the world (Ji et al., 2009). Recent evidence demonstrates their efficacy in some clinical scenarios. For example, the *Ginkgo biloba* extract EGb 760 attenuated neuronal loss in a mouse model of ischemic stroke and enhanced neurogenesis post-stroke (Nada et al., 2014). Furthermore, many natural compounds are available at low cost, allowing the possibility of relatively rapid transition to the clinical setting. We examined a library of natural compounds using the zebrafish (*Danio rerio*) lateral line as a model system to discover compounds that protect hair cells from aminoglycoside ototoxicity.

The zebrafish lateral line is an excellent model for high throughput screening of compounds that modulate hair cell survival, an intractable approach in mammalian systems (Harris et al., 2003; Owens et al., 2008; Coffin et al., 2013a,b). Lateral line hair cells are used to detect vibrations in the water for several behaviors, including predator avoidance and schooling (Partridge and Pitcher, 1980; Hoekstra and Janssen, 1983; Montgomery and Hamilton, 1997). These externally located hair cells are stereotypically organized into clusters known as neuromasts that are arrayed along the head and trunk of the fish (Metcalfe et al., 1985; Raible and Kruse, 2000). This external location provides an additional advantage, allowing for rapid access for pharmacologic manipulation similar to an *in vitro* preparation in an *in vivo* system. Zebrafish lateral line hair cells are structurally and functionally similar to mammalian hair cells. All vertebrate hair cells share core features, including an apical polarized hair bundle with mechanotransduction machinery (e.g., TMC proteins) and extracellular tip links composed of cadherin 23 and protocadherin 15 (Söllner et al., 2004; Kazmierczak et al., 2007; Pan et al., 2013; Maeda et al., 2014). Mutations in several of these proteins, for example the hair bundle motor protein Myosin VIIA, cause deafness in both mammals and zebrafish (Self et al., 1998; Ernest et al., 2000). Like inner ear hair cells, lateral line hair cells are bathed in a regulated ionic environment, with a gelatinous cupula overlying the apical hair bundles, similar to the cupula in the canal cristae of mammalian inner ears (Russell and Sellick, 1976; Valli et al., 1977; Van Netten, 1997). One key difference is that zebrafish hair cells, and indeed hair cells in all non-mammalian vertebrates, regenerate following ototoxic damage. This regeneration depends critically on supporting cells, rather than the hair cells themselves, such that while supporting cells differ between vertebrate groups, the hair cells are highly similar (Brignull et al., 2009). Importantly, fish hair cells respond similarly to toxic insult, making it likely that otoprotectants found in zebrafish studies will translate to mammals (Harris et al., 2003; Nicolson, 2005; Ton and Parng, 2005; Ou et al., 2007, 2009; Owens et al., 2008; Esterberg et al., 2013). For example, prior screening efforts demonstrated that the small molecule PROTO1 and the acetylcholinesterase inhibitor tacrine protect both zebrafish and mammalian hair cells from neomycin damage, highlighting the tractability of using zebrafish to discover otoprotective drugs (Owens et al., 2008; Ou et al., 2009). Using the zebrafish lateral line, we screened a natural compound library and discovered four novel compounds that protect hair cells from aminoglycoside exposure.

## Materials and Methods

### Animals

Zebrafish were maintained in zebrafish facilities at both Washington State University Vancouver and Amherst College. All cell biology experiments were conducted on 5–6 day old larval zebrafish (*AB strain, brn3c:mGFP (*Tg(pou4f3:gap43-GFP)*), or myo6b:EGFP (*Tg(myo6b:EGFP*))) maintained at 28.5°C in defined E2 embryo medium (EM) containing 1 mM MgSO_4_, 120 μM KH_2_PO_4_, 74 μM Na_2_HPO_4_, 1 mM CaCl_2_, 500 μM KCl, 15 mM NaCl, and 500 μM NaHCO_3_ in distilled water at a pH of 7.2 (Westerfield, 2000). We selected this age range because hair cells in 5 day-old fish show mature responses to ototoxic insult, and the small fish size allows for high throughput screening of compounds in small volumes (Murakami et al., 2003; Santos et al., 2006; Owens et al., 2008; Vlasits et al., 2012). Microphonics experiments used wild-type Ekkwill zebrafish (Ekkwill Waterlife Resources), which also exhibit neomycin-induced hair cell death similar to that observed in *AB fish (Kruger, unpublished data). All procedures were approved by the appropriate Institutional Animal Care and Use Committee at Washington State University and Amherst College.

### Aminoglycoside Treatment

Neomycin or gentamicin were used to induce damage to hair cells. Neomycin (10 mg/ml) and gentamicin (50 mg/ml) were acquired from Sigma-Aldrich (St. Louis, MO, USA) and diluted in EM. Fish were incubated with either neomycin for 30 min (termed “acute exposure”), or gentamicin for 6 h (termed “continuous exposure”) at a range of concentrations from 50–400 μM. Exposed fish were then rinsed 4× with fresh EM. Neomycin treated fish were allowed to recover for 1 h prior to hair cell assessment, while gentamicin treated fish were assessed immediately. These acute and continuous exposure time courses were chosen because there are distinct, yet overlapping, cell death pathways activated by aminoglycosides (Owens et al., 2009; Coffin et al., 2013a,b). Therefore, we asked if natural compounds universally protected hair cells from aminoglycoside-induced death, or if protection was specific for neomycin or gentamicin.

### Natural Compound Screen

We conducted two independent screens of the Enzo Natural Product Library (502 compounds, Enzo Life Sciences) to identify compounds that protect hair cells from neomycin or gentamicin damage. These two aminoglycosides were used because they activate different hair cell death mechanisms, allowing us to proactively screen for drug-specific protectants (Owens et al., 2009; Vlasits et al., 2012; Coffin et al., 2013a,b). Prior to treatment with each natural compound, fish were incubated in a 3 μM solution of the nuclear dye Yo-Pro-1 (Life Technologies) for 30 min to label hair cells (Santos et al., 2006; Owens et al., 2008). Yo-Pro-1 is a non-toxic dye that is highly amenable to screens of this nature because the dye is retained for at least 24 h (Coffin et al., 2009; Thomas et al., 2015). Therefore, hair cells could be labeled prior to treatment, allowing for rapid post-treatment assessment without the need to move the animal or add additional dyes. Three to four fish were placed in each well of a glass bottom 24-well plate; 2–3 wells each were used for positive (neomycin or gentamicin-only) and negative (DMSO-only) controls. 0.2% DMSO served as a negative control because it is the vehicle used to dissolve the natural compounds. Eighteen to 20 wells received aminoglycoside and one of the 502 natural compounds. Fish were pretreated for 1 h with a natural compound (4 μg/ml), co-treated for 1 h with the natural compound and 200 μM neomycin, or co-treated for 6 h with the natural compound and 100 μM gentamicin. Our treatment paradigm results in comparable hair cell loss with either aminoglycoside (Owens et al., 2009; Coffin et al., 2013a,b). Weight by volume (mg/ml) was used here to describe the concentration of the compounds in the Natural Products library instead of molarity because the library is standardized at 2 mg/ml for each compound. However, compounds in the library each have different molecular weights and therefore different molarities.

After treatment, fish were anesthetized with 0.001% buffered 3-aminobenzoic acid ethyl ester methanesulfonate (MS-222, Argent Labs, Redmond, WA, USA) prior to the assessment of anterior lateral line neuromasts (aLL) using a fluorescent dissection microscope (Leica M165FC, Leica Microsystems, Buffalo Grove, IL, USA). Given the rapid nature of the screen, the fluorescent intensity of every aLL neuromast was assessed holistically at once, as opposed to scoring specific neuromasts. A score of 0–3 was assigned to each fish: 0 (no visible hair cells); 1 (dim hair cell fluorescence); 2 (slightly bright hair cells); or 3 (fully bright hair cells; modified from Chiu et al., 2008). All compounds with a score of 2 or greater were rescreened in duplicate, and compounds that were confirmed to be protective through rescreening were analyzed via a concentration-response analysis.

### Hair Cell Assessment

We used the mitochondrial dye 2-(4-(dimethylamino)styryl)-N-ethylpyridinium iodide (DASPEI) for the bulk of our hair cell assessment experiments because it specifically and robustly labels lateral line hair cells, making it the labeling method of choice for lateral line visualization in many fish species (Harris et al., 2003; Coffin et al., 2009; Owens et al., 2009; Van Trump et al., 2010; Brown et al., 2011). Eight to 15 fish per treatment were incubated in EM containing 0.005% DASPEI (Life Technologies, Grand Island, NY, USA) for 15 min, rinsed 3× with fresh EM and anesthetized in 0.001% buffered MS-222. The brightness of 10 anterior neuromasts (SO1, SO2, IO1-IO4, M2, MI1, MI2, O2; Raible and Kruse, 2000) was assessed at 50× magnification using a Leica M165F fluorescent dissecting microscope. A scoring protocol was used to rank each neuromast as: 0 (no labeling); 1 (moderate labeling); or 2 (bright labeling). Neuromast scores were summed for a total score of 0–20 per fish (Harris et al., 2003; Owens et al., 2009).

Since our fluorescence assessment is semiquantitative, we validated the protection from aminoglycoside-induced hair cell death observed with DASPEI assessment through direct hair cell counts using immunocytochemistry. Fish were euthanized with an overdose of MS-222 and fixed with 4% paraformaldehyde overnight at 4°C. Fish were then blocked in 1% goat serum in phosphate-buffered saline (PBS; Life Technologies) with 0.1% Triton-X (PBST; Sigma-Aldrich) at room temperature for 2 h. To label hair cells, fish were incubated in mouse anti-parvalbumin (EMD Millipore, Billerica, MA, USA), diluted 1:500 in PBST with 1% goat serum (Coffin et al., 2013a). Fish were then rinsed in PBST and labeled with either Alexa 488 or 568 goat anti-mouse secondary antibodies (Life Technologies; diluted 1:500 in PBST). Following 2–3 additional rinses in PBST, and then PBS, fish were stored in 1:1 PBS/glycerol at 4°C prior to assessment. Fish were visualized using a compound fluorescent microscope (Leica DMI4000 B or DMRB). Hair cell number was quantified in five neuromasts (IO1, IO2, IO3, M2, OP1) per fish, summed to calculate one value per animal, and averaged for each group. We further confirmed our hair cell counts using Brn3c:mGFP fish, which express membrane-bound GFP in all hair cells, and myo6b:EGFP fish, which express cytoplasmic GFP in all hair cells (Xiao et al., 2005; Namdaran et al., 2012; Suli et al., 2014). Hair cells were counted in live, anesthetized transgenic larvae or freshly fixed larvae as described above.

### Concentration-Response Analysis

Four compounds scored a three during rescreening and were selected for further analysis. To determine the optimally protective concentration (OPC) for each natural compound, zebrafish were pretreated for 1 h with a range of compound concentrations (0.5–50 μM). The fish were then co-treated with the natural compound and 200 μM neomycin (acute) or 100 μM gentamicin (continuous) using the aminoglycoside treatment protocol described above. To determine the effectiveness of each natural compound at protecting hair cells, fish were pretreated with the OPC of each natural compound, then co-treated with the natural compound and 50–400 μM neomycin or gentamicin (Harris et al., 2003; Coffin et al., 2009). To assess broad applicability of potential protectants to diverse ototoxins, we also asked if these natural compounds conferred protection from cisplatin damage. Fish were pretreated with a range of protectant concentrations (0.25–10 μM) for 1 h, followed by co-treatment with protectant and 500 μM cisplatin for 6 h. Protection was assessed using DASPEI scoring and further validated by direct counts of anti-parvalbumin-labeled or GFP+ hair cells (for wildtype and Brn3c transgenic larvae, respectively). Experiments were conducted in triplicate.

### Aminoglycoside Uptake

Gentamicin conjugated to the dye Texas Red (GTTR) was used to quantify uptake of aminoglycosides into hair cells (Steyger et al., 2003; Wang and Steyger, 2009), and FM 1-43FX was used as a proxy for transduction channel function (Gale et al., 2001). Both GTTR and FM 1-43 are reported to primarily enter hair cells via the mechanoelectrical transduction (MET) channel, thereby offering two independent means of assessing channel block; FM 1-43FX is specifically taken up by the MET channel after a very brief exposure time (Alharazneh et al., 2011; Vu et al., 2013). Zebrafish were treated with 50 μM GTTR (made according to Steyger et al., 2003) for 3 or 18 min or 1.5 μM FM 1-43FX (Molecular Probes, Eugene, OR, USA) for 30 s. The fish were then rinsed 3× in EM, euthanized, and fixed with 4% paraformaldehyde. The IO3 neuromast was imaged using a 20× dry objective with 5× digital zoom on a Leica SP8 laser scanning confocal microscope with the 552 nm laser and 550–630 nm detection for GTTR (peak detection 580 nm), or the 488 nm laser and 590–680 nm detection for FM 1-43FX (peak detection 620 nm); the laser power and gain were kept constant within each experiment. Image stacks were collapsed into maximum projection images for analysis. Neuromast regions of interest (ROIs) were used to quantify average fluorescent intensity of each region (neuromast fluorescence—background fluorescence). Fluorescent intensity was measured in arbitrary units (a.u.) using ImageJ. IO3 neuromasts were selected because they were used for previous hair cell assessment and were consistently visible in every fish.

### Washout Experiment

We conducted a washout experiment to determine if natural compound treatment before or after, but not during aminoglycoside exposure, was sufficient to confer protection. We used gentamicin for this experiment because short-term exposure to gentamicin results in increasing hair cell damage over several hours, even after antibiotic removal (Owens et al., 2009). Fish were treated with 200 μM gentamicin for 30 min, followed by a 5.5 h recovery period in EM or a natural compound, and then assessed with DASPEI. Natural compound (or an equal volume of DMSO) was present before, during, and/or after gentamicin treatment, in order to assess the relative timing of the protective effect.

### Cell Proliferation

It is possible that protective compounds promote hair cell regeneration, rather than preserving existing hair cells. To examine this possibility, we conducted a cell proliferation assay using bromodeoxyuridine (BrdU). Larvae were treated with a natural compound for 1 h, then co-treated with compound and 200 μM neomycin for 30 min, rinsed in fresh EM, and allowed to recover for 1 h. Ten millimolar BrdU (Sigma) was present for the entire 2.5 h time course. Larvae were then euthanized with an overdose of MS-222, fixed overnight in 4% paraformaldehyde at 4°C, and processed for anti-BrdU immunocytochemistry using the protocol in He et al. (2015). 4,6-diamidino-2-phenylindole (DAPI, Life Technologies) was used as a counter-label to visualize neuromast location. Using a Leica DMRB microscope we quantified the number of BrdU+ cells per neuromast (5 neuromasts per animal: IO1, IO2, IO3, M2, OP1, the same neuromasts that were used for hair cell counts).

### Microphonics

MET channel function was assayed by performing extracellular recordings of microphonic potentials from the primary neuromasts (L1, L2, L3, L4) of the posterior lateral line (Trapani and Nicolson, 2010). Larvae were anesthetized with 0.016% MS-222 and pinned down on a silicone-lined (Sylgard-184, Dow Corning, Midland, MI, USA) recording chamber using homemade tungsten pins. The neurotoxin α-Bungarotoxin (125 μM; Abcam, Cambridge, MA, USA) was injected into the heart to prevent interference from muscle movement. To wash out the MS-222, larvae were rinsed with normal extracellular solution (130 mM, NaCl, 2 mM KCl, 2 mM CaCl_2_, 1 mM MgCl_2_ and 10 mM 4-(2-hydroxyethyl)-1 piperazineethane-sulfonic acid). Neuromasts were deflected ~20° bidirectionally at 20 Hz for 200 ms with a sinusoidal current of water produced by a high-speed pressure clamp (50 mMHg maximum output, HSPC-1, ALA Scientific, Farmingdale, NY, USA). The water current was directed at a single neuromast with a waterjet micropipette, which was fabricated using a Flaming/Brown style pipette puller (Model P-1000, Sutter Instruments, Novato, CA, USA) to pull borosilicate capillary tubing (B150-110-10HP, Sutter Instruments) to a long micropipette that was then cleanly broken to produce a 30 μm diameter tip. The waterjet was aligned with the anteroposterior axis of the fish and positioned approximately 100 μm from the hair cell bundle with the bottom of the waterjet even with the top of the neuromast cupula. Waterjet position was confirmed using an upright epifluorescent microscope with a 40× water immersion objective (BX51-WI, Olympus, Olympus Center Valley, PA, USA). The recording electrode was also fabricated from glass capillary tubing (BF150-110-10HP, Sutter Instrument) and was positioned just adjacent to the base of the cupula at the level of the hair cell stereocilia. Microphonic potentials were sampled at 20 kHz with a 1 kHz low-pass filter using an Axon Instruments 200B amplifier (Molecular Devices, Sunnyvale, CA, USA) in current-clamp mode (*I* = 0) with 500× gain and were then amplified (100×) and filtered 100 Hz using a Model 440 amplifier (Brownlee Precision, Palo Alto, CA, USA). Data were acquired with an ITC-16 DAQ device and Patchmaster software (HEKA Elektronik). Microphonic traces in the figures represent an average of 200 consecutive sweeps collected for each recording. The average magnitude of the evoked microphonic potential (in microvolts) was calculated from the integral of the microphonic waveform divided by 0.21 s (microvolt • s/s). The 0.21 s interval was from stimulus onset (*t* = 150 ms) to 10 ms after the stimulus offset (*t* = 360 ms).

### Antibiotic Efficacy

Kirby-Bauer antibiotic efficacy tests were used to determine if the otoprotective natural compounds affect the ability of neomycin or gentamicin to inhibit bacterial growth (Clinical and Laboratory Standards, 2009). Six millimeter filter article discs (Fisher Scientific, Waltham, MA, USA) were soaked overnight in either DMSO (control), 2 μg/ml neomycin (Owens et al., 2008) or 1 μg/ml gentamicin (Cohen et al., 1991; Ferguson, 2008), the OPC of the natural compound, or the natural compound and aminoglycoside. We used the minimum inhibitory concentration (MIC) of each antibiotic; a standard approach for assessing antibiotic efficacy (Clinical and Laboratory Standards, 2009). The MIC assumes that if the compound of interest (in this case, the natural compound) does not interfere with antibiotic efficacy at the lowest effective antibiotic concentration, then that same compound will likely not affect efficacy of higher antibiotic doses. However, we also tested a 15 mg/ml neomycin concentration to rule out compound/antibiotic interactions at higher antibiotic concentrations. The discs were removed from solution and allowed to dry for 1 h prior to plating to reduce saturation. The next day, *E. coli* strain ATCC25922 was plated on a 150 mm diameter agar plate (Mueller Hinton Agar; Fisher Scientific), and the pretreated discs were spaced evenly on the plate. The plate was then inverted and incubated overnight at 37°C. Images were taken of each plate using a Leica M165F microscope, and the area of *E. coli* growth inhibition around each disc was measured using ImageJ.

### Data Analysis

Comparisons were made via one-way or two-way analysis of variance (ANOVA) or *t*-test with Bonferroni multiple comparison correction, as appropriate, using GraphPad Prism 6.0. Data are presented as mean ± SEM.

## Results

**Figure 1:**
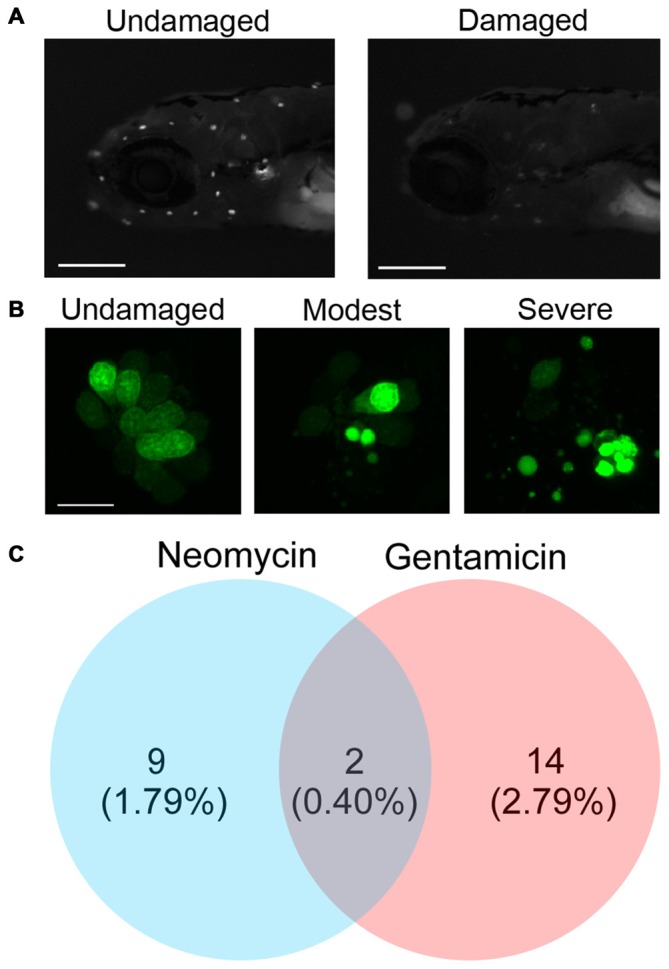
**Screening a natural compound library for otoprotective compounds. (A)** Examples of larval zebrafish labeled with the vital dye Yo-Pro-1. The left image is of an untreated fish, while the right image shows reduced neuromast fluorescence caused by exposure to 200 μM neomycin. Scale bars = 250 μm. **(B)** High magnification images of the SO2 neuromast from an untreated fish (left), fish with moderate hair cell damage (center), or fish with severe hair cell damage (right). Damage is evident by nuclear condensation, which results in more intense, punctate labeling, and by fragmentation. The scale bar on the left image = 10 μm and applies to all three panels. **(C)** Natural compounds protect zebrafish lateral line hair cells from the aminoglycosides neomycin and gentamicin. The initial screen produced nine compounds that protected hair cells from neomycin, 14 from gentamicin, and two from both ototoxins. The majority of the natural compounds screened (477) were not protective.

Using the zebrafish lateral line as a model of hair cell death, we screened a natural compound library for novel compounds that prevented neomycin- or gentamicin-induced hair cell death. Aminoglycoside treatment caused nuclear fragmentation and reduced neuromast fluorescence, while protective compounds largely prevented fragmentation and preserved labeling intensity (Figures 1A,B). From two independent screens, we found nine candidate natural compounds that protected hair cells from neomycin, 14 that protected from gentamicin, and two, hernandezine and decoyinine, that conferred protection from both aminoglycosides (Figure 1C). Overall, the percentage of otoprotective compounds that we initially detected from screening the Enzo Life Science Natural Product Library is comparable to similar otoprotection screens of other compound libraries (Ou et al., 2009; Vlasits et al., 2012).

**Figure 2:**
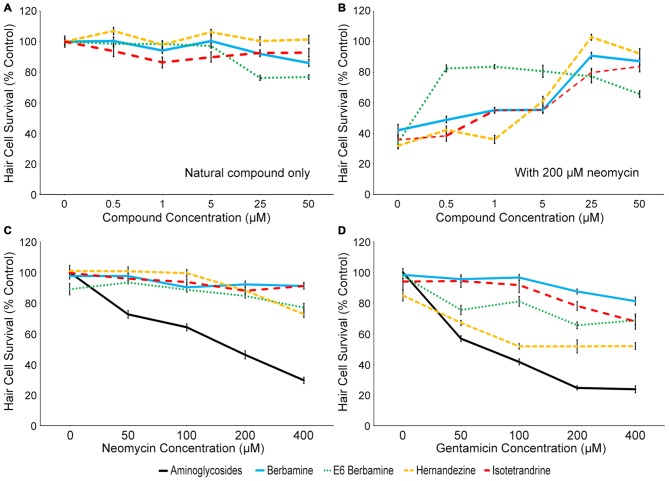
**Four natural compounds protect zebrafish lateral line hair cells from aminoglycoside-induced death. (A)** High concentrations of berbamine and E6 berbamine (25 or 50 μM) significantly damaged hair cells after 90 min of exposure (one-way ANOVA, berbamine: *F*_(5,84)_ = 11.75, *p* < 0.0001; E6 berbamine: *F*_(5,53)_ = 23.25, *p* < 0.0001). Iso also damaged hair cells, with significant loss at 1 μM (one-way ANOVA, *F*_(5,64)_ = 2.485, *p* = 0.0405). Her did not significantly damage hair cells (one-way ANOVA, *F*_(5,64)_ = 1.839, *p* = 0.1178) after 90 min. **(B)** Ber, E6, Her, and Iso robustly protect hair cells from acute 200 μM neomycin exposure, with optimal protective concentrations of 0.5 μM for E6 and 25 μM for Ber, Her, and Iso (one-way ANOVA, E6: *F*_(5,47)_ = 47.02, *p* < 0.001; Ber: *F*_(5,84)_ = 64.81, *p* < 0.0001; Her: *F*_(5,65)_ = 127.1, *p* < 0.0001; Iso: *F*_(5,59)_ = 56.59, *p* < 0.0001). **(C)** The optimally protective concentration (OPC) of Ber, E6, Her and Iso robustly protected hair cells from 50–400 μM acute neomycin exposure (two-way ANOVA, treatment effect, *F*_(4,317)_ = 212.2, *p* < 0.0001; interaction term *F*_(4,317)_ = 74.47, *p* < 0.0001). **(D)** All four compounds significantly protected hair cells from 50–400 μM continuous gentamicin exposure (two-way ANOVA, treatment effect, *F*_(4,281)_ = 263.8, *p* < 0.0001; interaction term *F*_(16,281)_ = 20.24, *p* < 0.0001). *N* = 8–26 and error bars are ±SEM.

Four compounds were selected for further evaluation (berbamine, E6 berbamine, hernandezine and isotetrandrine), because they consistently demonstrated 75% or greater otoprotection upon rescreening. We first determined whether 90 min of exposure to berbamine (Ber), E6 berbamine (E6), hernandezine (Her), or isotetrandrine (Iso) alone caused hair cell death (Figure 2A). We found that 25 and 50 μM of either Ber or E6 caused significant, albeit slight, damage to hair cells, with greater damage at 50 μM. We also found that 1 μM Iso slightly damaged hair cells (Figure 2A). However, all four compounds greatly protected hair cells from 200 μM neomycin. Ber, Her, and Iso were highly protective at 25 and 50 μM. In contrast, E6 was optimally protective at 0.5 μM, nearly 100-fold lower than the concentration that resulted in hair cell damage (Figure 2B). Based on these results, we selected the OPC of 0.5 μM for E6, and 25 μM for Ber, Her, and Iso, to further characterize the nature of protection. In order to determine the effectiveness of otoprotection, we asked if the OPC of each natural compound conferred protection from a range of aminoglycoside concentrations. All four natural compounds protected hair cells from 50–400 μM acute neomycin exposure (Figure 2C) and 50–400 μM continuous gentamicin exposure (Figure 2D). Overall, all four natural compounds offered protection from neomycin and gentamicin-induced hair cell death, albeit with some ototoxicity at higher concentrations.

**Figure 3:**
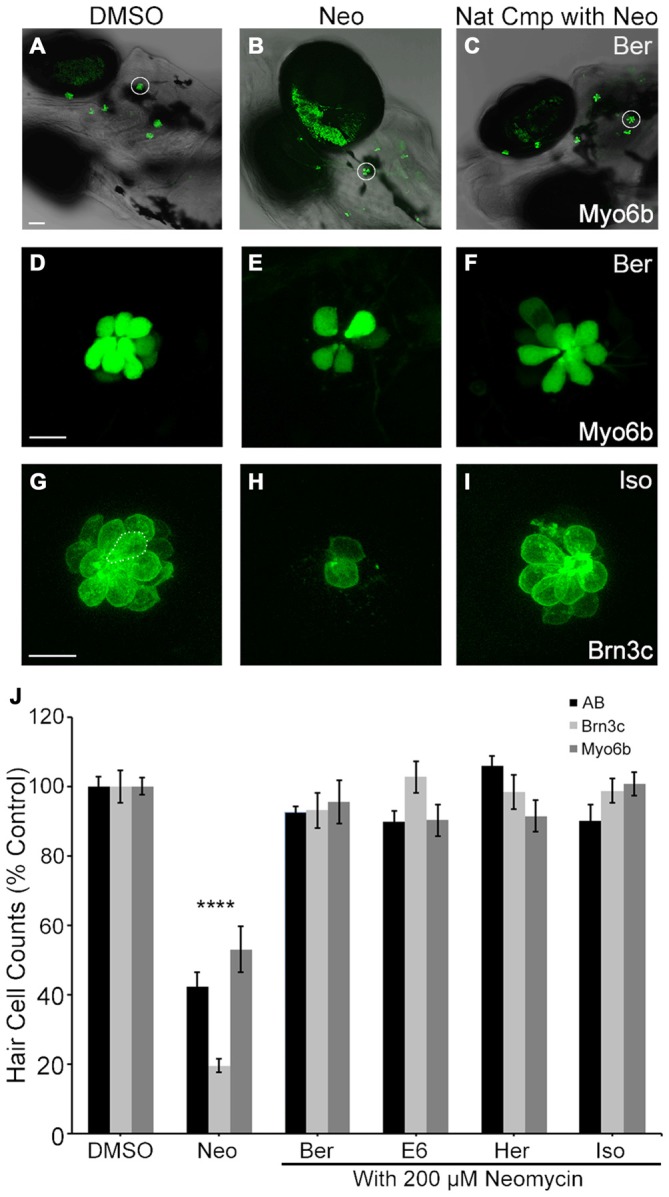
**Direct hair cell counts confirm otoprotection.** Ber, E6, Her and Iso robustly protect hair cells from 200 μM neomycin after acute exposure. **(A–F)** Representative low magnification **(A–C)** and high magnification **(D–F)** images of myo6b: EGFP transgenic fish treated with the indicated conditions. White circles in **(A–C)** denote the neuromasts shown in **(D–F)**. Scale bar in **(A)** = 40 μm and applies to panels **(A–C)**, scale bar in **(D)** = 10 μm and applies to panels **(D–F)**. **(G–I)** Representative images of brn3c: mGFP hair cells treated with the indicated conditions. **(G)** The dotted line encircles a hair cell. Scale bar in **(G)** = 10 μm and applies to panels **(G–I)**. **(J)** Direct counts of hair cells in five neuromasts per fish demonstrate significant hair cell protection in *AB fish labeled with anti-parvalbumin (black bars, one-way ANOVA, *F*_(4,60)_ = 46.58, *p* < 0.0001), Brn3c fish (light gray bars, one-way ANOVA, *F*_(4,38)_ = 75.58, *p* < 0.0001), and myo6: EGFP fish (dark gray bars, one-way ANOVA, *F*_(4,53)_ = 14.86, *p* < 0.0001). *N* = 8–24 and error bars are ±SEM. and ****p < 0.0001.

Our primary assessment of hair cell viability used DASPEI labeling, where fluorescent intensity is dependent on mitochondrial potential (Bereiter-Hahn, 1976). However, it is possible that changes in mitochondrial potential do not accurately reflect hair cell death. Therefore, to directly confirm the protection observed using DAPSEI scoring, we also counted the number of viable hair cells remaining after treatment in immunocytochemically-labeled wild type larvae, transgenic brn3c:mGFP larvae, and transgenic myo6b:EGFP larvae. All validation measures clearly demonstrated that all four natural compounds protected hair cells from 200 μM acute neomycin exposure, with neomycin alone killing over 50% of the hair cells and each compound conferring approximately 90% protection (Figure 3). The results of these independent assessments confirm the hair cell protection observed and further validate the strong correlation between DASPEI scoring and hair cell counts (Harris et al., 2003; Coffin et al., 2013a).

**Figure 4:**
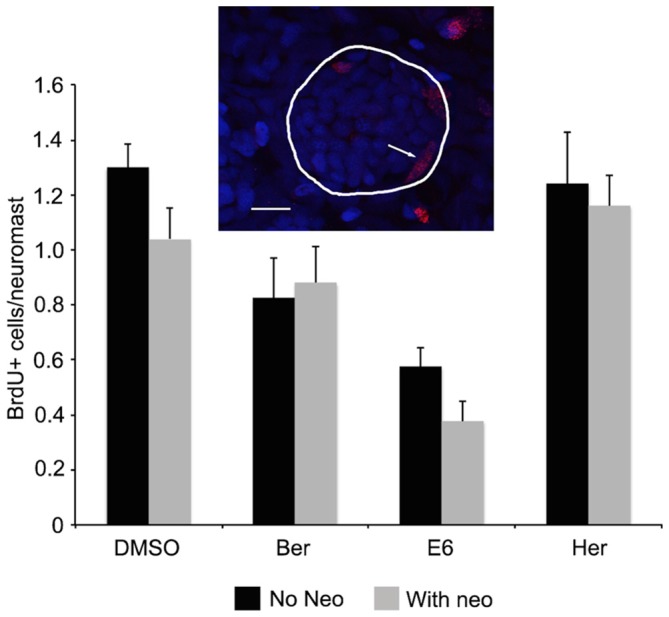
**Ber, E6, and Her do not enhance regeneration rates after neomycin damage.** Fish were treated with either natural compound only or natural compound and 200 μM neomycin in the presence of BrdU. Fish were counterstained with DAPI to identify neuromasts based on characteristic morphology, as shown in the inset where DAPI-labeled nuclei are blue and red nuclei are BrdU+. The line encircles the neuromast and the arrow denotes a BrdU+ supporting cell at the neuromast periphery. Scale bar = 10 μm. There is no difference in the number of BrdU+ cells per neuromast in fish that received neomycin + compound (gray bars) vs. those that received compound only (black bars; 2-way ANOVA, treatment effect *F*_(1,67)_ = 1.882, *p* = 0.175). *N* = 8–10 and bars are ±SEM.

The lateral line of larval zebrafish rapidly regenerates lost hair cells, with complete regeneration observed 72 h after neomycin damage (Harris et al., 2003; Ma et al., 2008). We therefore wanted to rule out that we were observing a combination of hair cell protection and increased hair cell regeneration. Following neomycin damage and 1 h of recovery, neuromasts had an average of 1.0 ± 0.1 BrdU+ cells per neuromast, and this number did not increase upon co-treatment with Ber, E6, or Her (0.9 ± 0.1, 0.4 ± 0.1, 1.2 ± 0.1 BrdU+ cells per neuromast, respectively, Figure 4). These data demonstrate that our bisbenzoquinoline derivatives protect hair cells from aminoglycoside damage, rather than promoting rapid hair cell regeneration.

### Natural Compounds Protect Hair Cells by Reducing Uptake of Aminoglycoside Antibiotics

**Figure 5:**
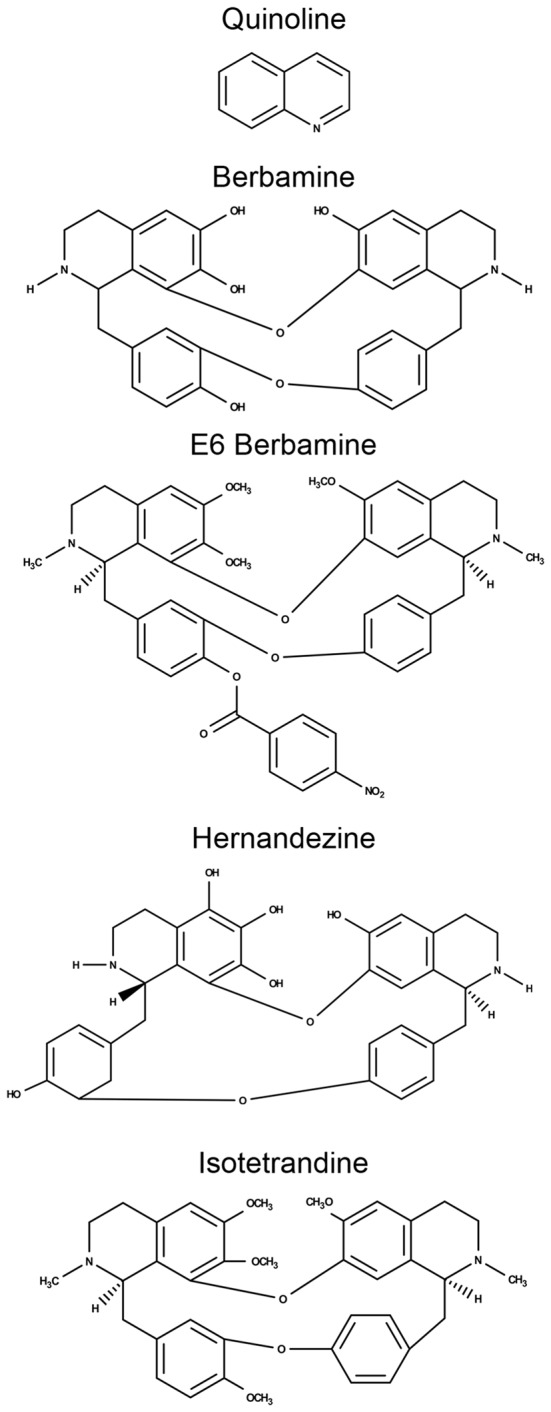
**Comparison of their chemical structures shows that all four natural compounds contain modified quinolone rings**.

**Figure 6:**
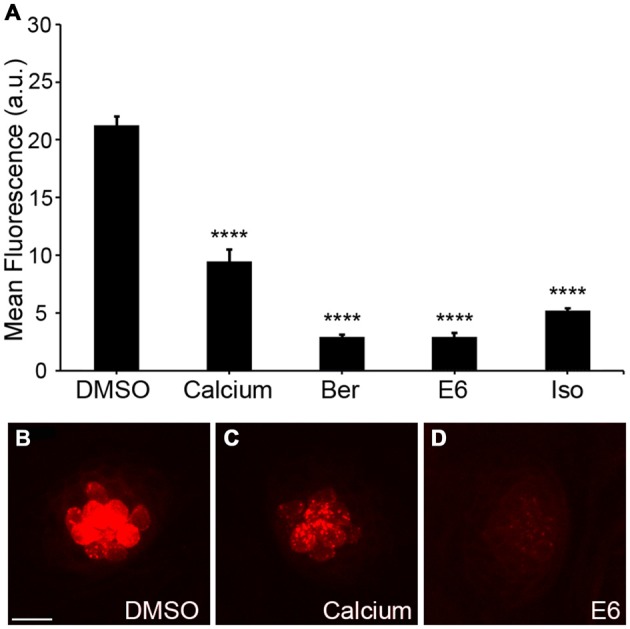
**Natural compounds reduce uptake of gentamicin conjugated with Texas Red (GTTR). (A)** The OPC of Ber, E6, or Isosignificantly reduced fluorescent intensity of 50 μM GTTR after 18 min of GTTR incubation (one-way ANOVA, *F*_(4, 69)_ = 179.0, *p* < 0.0001). Fluorescent intensity was measured in arbitrary units (a.u.). **(B–D)** Representative images of 18 min GTTR uptake in DMSO (negative control), high Ca^2+^ (positive control), and E6. Scale bar in **(B)** = 10 μm and applies to all images. *N* = 13–15 and bars are +SEM. and ****p < 0.0001.

Aminoglycosides rapidly enter hair cells via MET channels that are located at the apical portion of the hair bundle (Alharazneh et al., 2011; Vu et al., 2013). Ou et al. (2012) showed that quinoline ring derivatives reduce uptake of aminoglycosides into hair cells, and all four of our protective compounds contain modified quinoline ring structures (bisbenzylisoquinoline rings; Figure 5). As such, we hypothesized that these natural compounds confer protection by reducing aminoglycoside access to hair cells through the MET channel. To test this hypothesis, we used gentamicin conjugated to Texas Red (GTTR) to quantify uptake of gentamicin into hair cells (Steyger et al., 2003). Application of 25 μM Ber, 0.5 μM E6, or 25 μM Iso significantly reduced uptake of 50 μM GTTR after 18 min of incubation (Figure 6). A similar reduction in uptake was seen after 3 min of incubation in GTTR (data not shown). We elected to remove Her from further investigation because it is 10-fold more expensive than the other natural compounds and is lethal to zebrafish after prolonged exposure (data not shown). For the GTTR experiment, Ca^2+^ was used as a positive control since increasing the calcium concentration decreases the probability of opening the MET channel, reducing the amount of GTTR that enters hair cells (Ricci and Fettiplace, 1998; Coffin et al., 2009).

**Figure 7:**
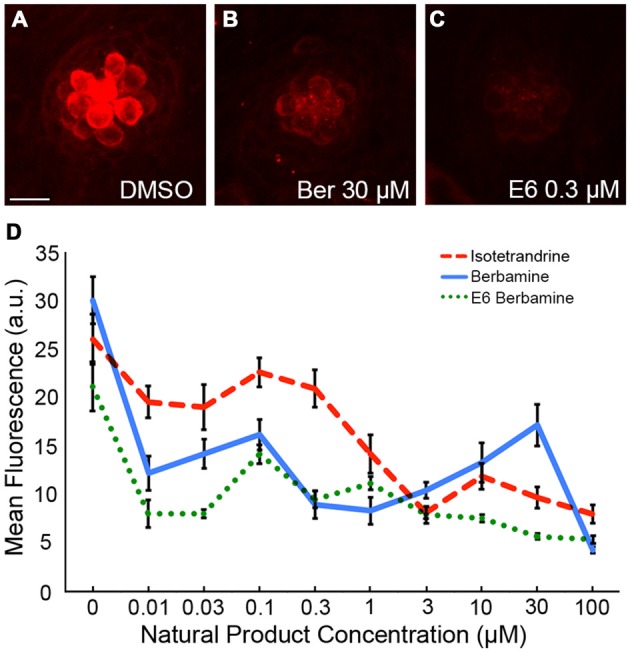
**Natural compounds reduce FM 1-43FX entry into hair cells. (A–C)** Representative images showing a reduction in FM 1-43FX uptake with Ber and E6. The scale bar in **(A)** = 10 μM and applies to all images. **(D)** Ber, E6, and Iso reduce uptake of FM 1-43FX into hair cells in a dose-dependent manner. Fluorescent intensity was measured in arbitrary units (a.u.). Data were analyzed by one-way ANOVA for each compound, with *p* < 0.0001 in all cases. *N* = 10–14 and bars are ±SEM.

**Figure 8:**
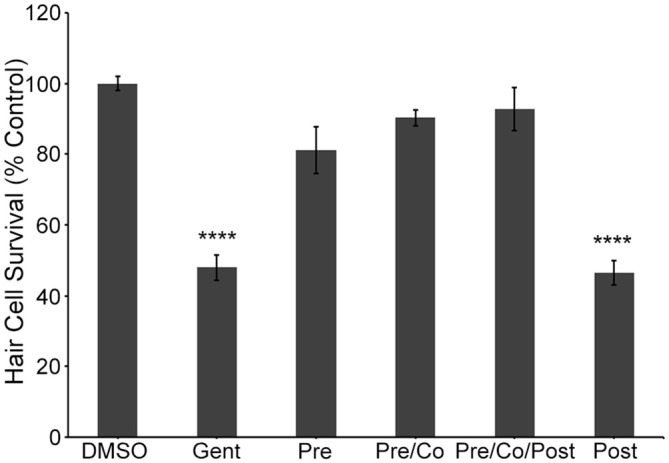
**Iso protection requires pre and/or co-treatment with natural compound and aminoglycoside.** Protection from gentamicin damage is evident if the natural compound is present before (Pre) and during gentamicin exposure (Pre and Co) regardless of natural compound presence during the post-treatment recovery period (Post). However, the presence of natural compound during the post-treatment recovery period only (Post) is not sufficient to confer protection. These data suggest that these natural compounds do not protect hair cells by modulating an intracellular signaling pathway (one-way ANOVA, *F*_(5,79)_ = 56.86, *P* < 0.001). *N* = 9–22, bars are ±SEM., ****p < 0.0001.

**Figure 9:**
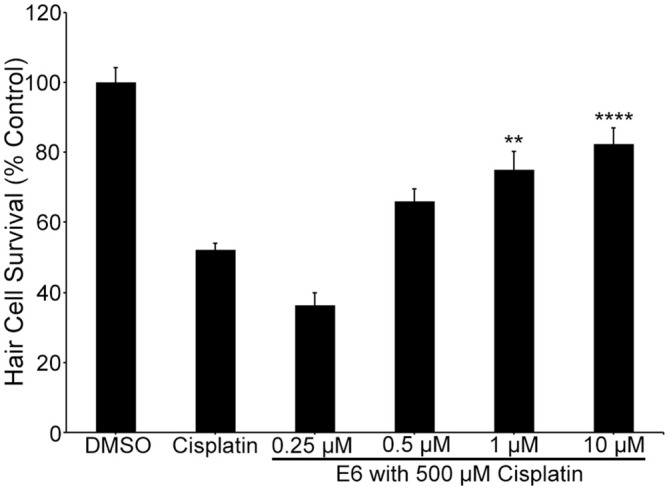
**E6 attenuates cisplatin ototoxicity.** One and 10 μM E6 protects hair cells from 6 h of exposure to 500 μM cisplatin (one-way ANOVA, *F*_(5,73)_ = 33.48, *p* < 0.0001). *N* = 12–15 and bars are +SEM. **p < 0.01, ****p < 0.0001.

To independently validate our hypothesis that the natural compounds provide protection by blocking uptake through the MET channels, we also used the fluorescent vital dye, FM 1-43FX, which rapidly increases in fluorescence upon entering a hair cell through the MET channels (Gale et al., 2001; Meyers et al., 2003). Log and half-log concentrations (0.01–100 μM) of E6, Ber, and Iso reduced FM 1-43FX uptake in a concentration-dependent manner (Figure 7). Additionally, a washout experiment with gentamicin showed that protection was abolished if zebrafish were removed from gentamicin before adding Iso, suggesting a transient inhibitory mechanism consistent with an uptake block (Figure 8). In contrast, pre-treatment with Iso conferred significant, albeit reduced, protection from gentamicin toxicity, suggesting that the uptake block may be maintained after compound removal (Figure 8). Similar results were seen in washout experiments with Ber and E6 (data not shown). Lastly, we asked if E6 protected hair cells from cisplatin, another ototoxin that enters hair cells through the MET channel (Thomas et al., 2013). We elected to focus on E6 for the cisplatin experiments because all four natural compounds have similar structures and appear to confer protection via similar mechanisms. Furthermore, since DMSO complexes with cisplatin to increase ototoxic damage, we selected the natural compound with lowest OPC, which also equates to the lowest concentration (0.002%) of DMSO (Uribe et al., 2013). Following 6 h exposure to 500 μM cisplatin, cotreatment with 0.5 μM E6 did not confer protection from hair cell death, but 1 and 10 μM E6 were significantly protective (Figure 9). Collectively, these data support our hypothesis that these bisbenzylisoquinoline-containing alkaloids protect hair cells by reducing uptake of chemical ototoxins through the MET channel.

### Protected Hair Cells are Still Functional after Exposure to E6 Berbamine

To determine whether E6 could elicit a protective effect against aminoglycoside ototoxicity without altering hair cell function, we assessed MET channel function using microphonic recordings. The MET channel is a non-selective cation channel that opens upon mechanical deflection of the hair bundle (Howard et al., 1988). When neuromast hair bundles are deflected, inward potassium and calcium currents through the MET channel result in depolarization of the receptor potential and a hyperpolarization of extracellularly-recorded microphonic potentials (Corey and Hudspeth, 1983; Nicolson et al., 1998).

**Figure 10:**
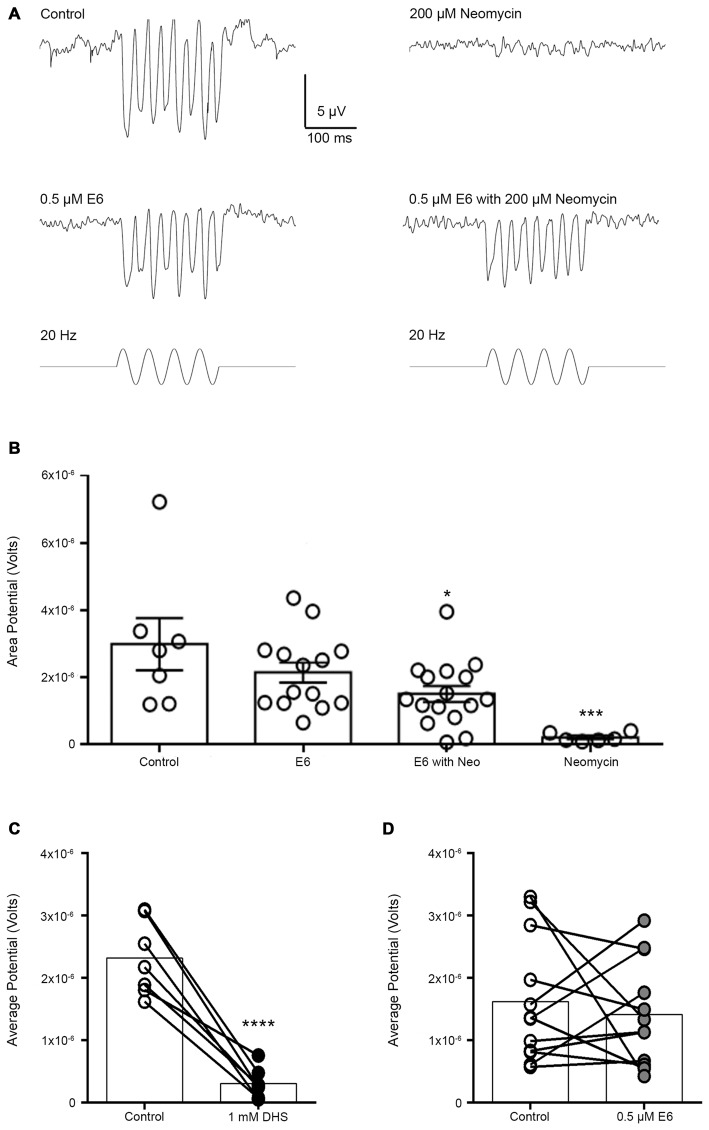
**Co-treatment with E6 berbamine protects hair cells from neomycin-induced loss of microphonics. (A)** Representative microphonic traces from control, E6, E6 + Neo, and neomycin-treated hair cells during 20 Hz stimulation for 200 ms. Treatment with 200 μM neomycin alone results in a loss of microphonic potentials. **(B)** Microphonic potentials for control and E6 only group are not significantly different from one another (one-way ANOVA, multiple comparisons, *p* > 0.05). However, there was a significant difference between the control and the E6 with neo group, where hair cells treated with E6 + neo had significantly reduced microphonics compared to control or E6-only-treated cells (one-way ANOVA, multiple comparisons, *p* < 0.05). Furthermore, E6 prevents the loss of microphonic potentials normally seen with neomycin (one-way ANOVA, *F*_(3,39)_ = 6.609, *p* = 0.001). **(C)** Acute application of 1 mM dihydrostreptomycin (DHS) significantly reduces microphonic potentials by nearly 87% (paired *t*-test, *p* < 0.0001). **(D)** Acute application of E6 does not affect microphonic potentials compared to control (paired *t*-test, *p* > 0.05). *N* = 6–16 and bars are ±SEM, *p < 0.05, ***p < 0.001, and ****p < 0.0001.

Consistent with hair cell damage, neomycin treatment caused a significant reduction in microphonic potentials evoked by 200 ms of 20-Hz sinusoidal stimulation (Figure 10A). In contrast, hair cells co-treated with E6 and neomycin had robust, albeit significantly reduced, microphonic potentials, indicating that many of the E6-protected hair cells remained functional (Figure 10B). To assay whether E6 is able to acutely block hair cell transduction at the concentrations used for our protection experiments, we applied 0.5 μM E6 to the bath while recording microphonics. Direct application of E6 did not reduce microphonic potentials. As a positive control, we then confirmed acute block of microphonics with 1 mM dihydrostreptomycin (DHS), a known MET blocker (Marcotti et al., 2005), which reduced microphonic potentials significantly (Figures 10C,D). Increasing E6 to a concentration (25 μM) that significantly blocked FM 1-43X uptake also had no effect on microphonic potentials (*p* = 0.2), nor did application of 25 μM Iso (*p* = 0.9). Overall, these electrophysiological results demonstrate that these bizbenzoquinoline derivatives protected lateral line hair cells from aminoglycoside toxicity without affecting their function, making them candidates for therapeutic development.

### Protective Compounds do not Interfere with Aminoglycoside Antibiotic Efficacy

**Figure 11:**
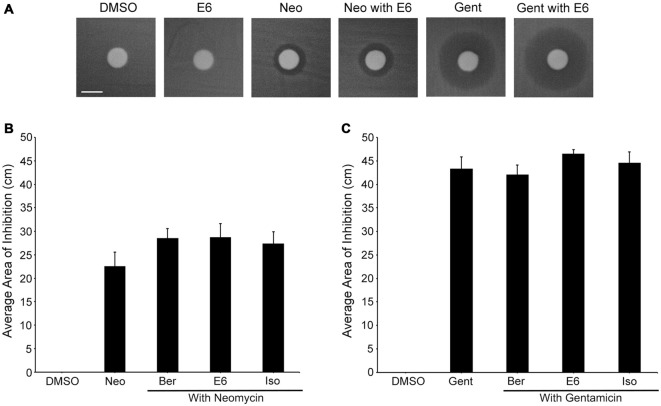
**Ber, E6, and Iso do not interfere with aminoglycoside efficacy. (A)** Representative images show no obvious difference between neomycin-only and neomycin with E6 or gentamicin-only and gentamicin with E6 groups; none of the natural compounds alone demonstrate antibacterial activity. Scale bar in **(A)** = 0.6 cm and applies to all images. **(B–C)** Measuring the area of the zone of inhibition show that none of the natural compounds significantly affect the area of inhibition of either **(B)** Neomycin (one-way ANOVA, *F*_(3,20)_ = 1.256, *p* = 0.3162) or **(C)** Gentamicin (one-way ANOVA, *F*_(3,19)_ = 0.9563, *p* = 0.4335). *N* = 6 and bars are +SEM.

To determine if E6, Ber, or Iso altered antibiotic efficacy, we conducted a Kirby-Bauer agar diffusion test (Clinical and Laboratory Standards, 2009). We co-treated *E. coli* with the optimal protective concentration of each natural compound and the MIC of neomycin (2 μg/ml) or gentamicin (1 μg/ml) and measured the area of inhibited growth. None of the natural compounds affected neomycin’s or gentamicin’s ability to inhibit growth of *E. coli* strain ATCC25922 (Figure 11). Furthermore, none of the natural compounds alone inhibited bacterial growth. We observed similar results using 15 mg/ml neomycin. The higher neomycin concentration yielded a growth inhibition area of 34.0 ± 2.3 cm, while neomycin plus E6, Her, or Iso lead to inhibition areas of 35.2 ± 2.2, 34.9 ± 1.9, and 36.3 ± 1.7 cm, respectively.

## Discussion

Our initial independent otoprotection screens using neomycin and gentamicin detected 23 natural compounds that protected hair cells from ototoxic damage. To weed out false positives, the 23 natural compounds were rescreened in duplicate. We discovered four bisbenzylisoquinoline compounds from the two screens, berbamine, E6 berbamine, hernandezine, and isotetrandrine, that consistently protected hair cells from acute neomycin and continuous gentamicin exposure paradigms; the other 19 natural compounds were not protective upon repeated testing. All four protective compounds substantially reduced GTTR and FM 1-43FX uptake, two compounds that enter hair cells via the MET channel. Electrophysiological recordings of microphonic potentials confirmed that natural compounds alone do not disrupt hair cell function and also serve to preserve hair-cell transduction following ototoxic insult. To our knowledge this is the first time that a library of natural compounds has been screened for otoprotection.

Our results suggest that these bisbenzylisoquinoline-containing natural compounds protect hair cells by blocking aminoglycoside uptake through the MET channel, which is the primary site of aminoglycoside entry in hair cells (Marcotti et al., 2005; Alharazneh et al., 2011; Vu et al., 2013). However, other routes of aminoglycoside entry have been reported, raising the possibility that these natural compounds act by blocking one of the alternative uptake mechanisms (Huth et al., 2011). One alternative is that these natural compounds interfere with receptor-mediated endocytosis, since kanamycin was shown to colocalize with cationic ferritin (a marker for receptor-mediated endocytosis) in regions immediately below the apical plasma membrane of chicken sensory hair cells (Hashino and Shero, 1995). It is also possible that these natural compounds are acting as antioxidants to protect hair cells, since isoquinoline alkaloids have some antioxidant abilities (Zarei et al., 2015). However, this antioxidant hypothesis seems unlikely as no protection was observed in fish treated with natural compounds *after* gentamicin exposure, a time when cell death is occurring. The uptake block may be temporary, as removing the natural compound prior to treatment with gentamicin attenuated protection. However, some protection was retained in our washout study, suggesting that partial block may be maintained. All four otoprotective compounds have modified quinoline-like ring structures, and previous studies demonstrate that other quinoline ring derivatives, such as the FDA approved bioactive compounds tacrine and amsacrine, also block uptake of aminoglycosides into hair cells (Ou et al., 2009, 2012). The unique structure of these natural compounds may provide new information about how quinoline rings attenuate aminoglycoside uptake. For example, E6 has an additional benzene ring with an attached nitrite group that may increase its affinity for the MET channel (or other uptake receptor) compared to Ber, consistent with E6 acting at a substantially lower concentration. It is possible that the quinoline rings structures are not necessary to confer protection, and future experiments will examine a suite of chemical analogs to determine the minimum chemical structure that confers otoprotection. Whether or not the modified quinoline ring structures are necessary, our data demonstrate that these compounds protect hair cells by preventing aminoglycoside entry. By temporarily blocking ototoxin uptake, the mode of action of these natural compounds may be an appealing way to prevent aminoglycoside-induced hair cell death, as modulating specific intracellular signaling pathways is often complex and may result in unintended consequences for non-target cells. The nature of the relationship between these bis-benzoquinoline derivatives and aminoglycoside uptake mechanisms offers an avenue for future exploration.

As stated above, E6 was chosen for additional study because it is relatively inexpensive and had the lowest optimal protective concentration of the four compounds and of any other quinoline derivative found to date, suggesting a greater potency (Ou et al., 2012). Although E6 reduced uptake of GTTR and FM 1-43FX, microphonic recordings from lateral line hair cells showed that E6-treatment alone did not disrupt normal hair cell transduction, with hair cells previously exposed to E6 and neomycin displaying robust microphonic recordings. Additionally, acute application of E6 did not significantly reduce microphonics, suggesting that if E6 protection is conferred by direct interaction with the MET channel, it is likely not disrupting cation flow through the channel. Consistent with an interaction with the MET channel, E6 application was able to block larger molecules, such as neomycin (614 Da), gentamicin (477 Da), GTTR (1165 Da) and FM 1-43FX (611 Da), which are presumably unable to enter the partially obscured channel. An alternative possibility is that the natural compounds have a faster on/off rate than the aminoglycosides, allowing them to outcompete the larger molecules for access to the channel during cotreatment. However, this competitive inhibition should have resulted in reduced microphonics during acute application of E6 at concentrations that blocked FM 1-43X uptake. Another possibility is that these compounds reduce aminoglycoside uptake by a more indirect interaction with the MET channel similar to amiloride (Jørgensen and Ohmori, 1988). Alternatively, these compounds could cause modulation of intracellular calcium which may alter the MET channel open probability, since quinoline derivatives have been shown to block calcium influx associated with depleted calcium pools in bovine endothelial cells (Low et al., 1996; Ricci and Fettiplace, 1998; Eatock, 2000). However, this alternative explanation is unlikely since we did not see a reduction in the microphonic potentials, which would be expected with changes in calcium dynamics (Nicolson et al., 1998).

Cisplatin also enters hair cells through the MET channel (Thomas et al., 2013), but 0.5 μM E6 did not protect hair cells from cisplatin toxicity, while this concentration confers essentially complete protection from aminoglycoside damage. However, increasing the concentration of E6 to 1 or 10 μM significantly protected hair cells from cisplatin toxicity. This result is not surprising given that cisplatin molecules (300 Da) are smaller than the larger aminoglycoside compounds. Future research is needed to determine how these otoprotective natural compounds interact with the MET channel or other antibiotic entry route to reduce aminoglycoside uptake.

Other investigators have used the zebrafish lateral line to discover otoprotective compounds. Vlasits et al. (2012) screened a library of 640 FDA-approved compounds and identified 10 compounds that protected from acute neomycin exposure, with only seven of those 10 compounds protecting from continuous gentamicin exposure. It is not surprising that the Vlasits screen found more otoprotective compounds, as all of the compounds in the FDA library were known to activate specific cellular pathways. Similarly, a screen of a library containing 1040 biologically active compounds identified seven compounds that prevented neomycin ototoxicity (Ou et al., 2009). Screening a library of 10,960 small molecules revealed two benzothiophene carboxamide compounds (PROTO-1 and PROTO-2) that protected hair cells from acute neomycin exposure (~0.02% hit rate), but not continuous gentamicin exposure (Owens et al., 2008). Neomycin and gentamicin activate overlapping, yet distinct, cell death pathways in zebrafish hair cells (Owens et al., 2009; Coffin et al., 2013a,b). Therefore, it was not unexpected that some screens found compounds that protect from one antibiotic or the other, suggesting that these compounds modulate specific intracellular pathways. However, all four compounds from the present screen conferred protection from both neomycin and gentamicin, suggesting that these natural compounds may be broadly applicable to prevent aminoglycoside ototoxicity.

Ber, Her, and Iso are bisbenzylisoquinoline alkaloids from the *Thalictrum* family (herbaceous perennial flowering plant; Schif, 1991; Pelletier, 1996); Ber, and Iso are also found in *Berberis*, a shrub (Weber and Fournet, 1989; Di et al., 2003). E6 berbamine is a synthetic derivative of berbamine. None of these natural compounds have been reported to affect hearing, and since they are not well studied, there is little information about bioavailability or serum concentrations in mammals. However, all four natural compounds have been studied to treat various disorders, including cancer and pain; bisbenzylisoquinoline alkaloids have anti-tumor properties (Kuroda et al., 1976) and Ber reduces inflammation and pain responses in mice with serotonin-induced hind paw edema (Küpeli et al., 2002). This research shows the promising applications that natural compounds have in treating a wide variety of medical conditions, and our data add hair cell protection to this growing list of therapeutic possibilities.

Our otoprotective alkaloids did not affect antibiotic efficacy of neomycin or gentamicin, making them ideal for future research in mammalian systems, and ultimately as potential therapeutics to prevent hearing loss in humans. One caveat of using the zebrafish lateral line as an ototoxicity model is the rapid rate of hair cell regeneration, where newly generated hair cells may be confused with protected hair cells. However, aminoglycoside-induced hair cell death occurred within hours of antibiotic application, whereas substantial regeneration is only evident 48 h after hair cell death (Harris et al., 2003; Ma et al., 2008). We did not observe an increase in cell proliferation after co-administration of natural compound and neomycin, consistent with our interpretation that our natural compounds confer true hair cell protection. In addition to determining if Ber, E6, and Iso protect mammalian hair cells, future work will include using the structural similarities of the four natural compounds to create more efficient otoprotective synthetic derivatives. For example, the synthetic berbamine, E6, protects hair cells at a lower concentration than Ber, Iso, or Her. Natural compounds offer a rich source of functionally and chemically diverse otoprotective compounds with the potential to reduce the ototoxic side effect of a highly effective class of antibiotics.

## Author Contributions

Participated in research design: MK, AJO, JGT, ABC. Conducted experiments: MK, RB, AJO, TFS, JGT, ABC. Performed data analysis: MK, JT, AC. Wrote or contributed to the writing of the manuscript: MK, RB, AJO, TFS, JGT, ABC.

## Conflict of Interest Statement

The authors declare that the research was conducted in the absence of any commercial or financial relationships that could be construed as a potential conflict of interest. The handling Editor declares a current collaboration with the authors and states that the process nevertheless met the standards of a fair and objective review.

## References

[B1] Alharazneh A. Luk L. Huth M. Monfared A. Steyger P. S. Cheng A. G.. (2011). Functional hair cell mechanotransducer channels are required for aminoglycoside ototoxicity. PLoS One 6:e22347. 10.1371/journal.pone.0022347 21818312 PMC3144223

[B2] Bereiter-Hahn J. (1976). Dimethylaminostyrylmethylpyridiniumiodine (daspmi) as a fluorescent probe for mitochondria in situ. Biochim. Biophys. Acta 423, 1–14. 10.1016/0005-2728(76)90096-7 764877

[B3] Blackwell D. L. Lucas J. W. Clarke T. C. (2014). Summary health statistics for U.S. adults: national health interview survey, 2012. National center for health statistics. Vital Health Stat. 10, 1–171. 24819891

[B4] Brignull H. R. Raible D. W. Stone J. S. (2009). Feather and fins: non-mammalian models for hair cell regeneration. Brain Res. 1277, 12–23. 10.1016/j.brainres.2009.02.028 19245801 PMC2700174

[B5] Brown A. D. Mussen T. D. Sisneros J. A. Coffin A. B. (2011). Reevaluating the use of aminoglycoside antibiotics in behavioral studies of the lateral line. Hear. Res. 272, 1–4. 10.1016/j.heares.2010.10.014 21055458 PMC3114637

[B6] Chiu L. L. Cunningham L. L. Raible D. W. Rubel E. W. Ou H. C. (2008). Using the zebrafish lateral line to screen for ototoxicity. J. Assoc. Res. Otolaryngol. 9, 178–190. 10.1007/s10162-008-0118-y 18408970 PMC2504598

[B7] Clinical and Laboratory Standards (2009). Methods for Dilution Antimicrobial Susceptibility Tests for Bacteria that Grow Aerobically; Approved Standard. (Vol. 29), 8th Edn. Wayne, PA: Clinical and Laboratory Standards Institute.

[B8] Coffin A. B. Reinhart K. E. Owens K. N. Raible D. W. Rubel E. W. (2009). Extracellular divalent cations modulate aminoglycoside-induced hair cell death in the zebrafish lateral line. Hear. Res. 253, 42–51. 10.1016/j.heares.2009.03.004 19285547 PMC5139914

[B9] Coffin A. B. Rubel E. W. Raible D. W. (2013a). Bax, Bcl2 and p53 differentially regulate neomycin- and gentamicin-induced hair cell death in the zebrafish lateral line. J. Assoc. Res. Otolaryngol. 14, 645–659. 10.1007/s10162-013-0404-1 23821348 PMC3767879

[B10] Coffin A. B. Williamson K. L. Mamiya A. Raible D. W. Rubel E. W. (2013b). Profiling drug-induced cell death pathways in the zebrafish lateral line. Apoptosis 18, 393–408. 10.1007/s10495-013-0816-8 23413197 PMC3627356

[B11] Cohen M. A. Huband M. D. Mailloux G. B. Yoder S. L. (1991). *In vitro* antibacterial activities of the fluoroquinolones PD 117596, PD 124816 and PD 127391. Diagn. Microbiol. Infect. Dis. 14, 245–258. 10.1016/0732-8893(91)90039-i 1889177

[B12] Corey D. P. Hudspeth A. J. (1983). Analysis of the microphonic potential of the bullfrog’s sacculus. J. Neurosci. 3, 942–961. 6601693 10.1523/JNEUROSCI.03-05-00942.1983 PMC6564510

[B13] Di D. Liu Y. Ma Z. Jiang S. (2003). Determination of four alkaloids in *Berberis* plants by HPLC. China J. Chin. Mater. Med. 28, 1132–1134. 15617491

[B14] Durante-Mangoni E. Grammatikos A. Utili R. Falagas M. E. (2009). Do we still need the aminoglycosides? Int. J. Antimicrob. Agents 33, 201–205. 10.1016/j.ijantimicag.2008.09.001 18976888

[B15] Eatock R. A. (2000). Adaptation in hair cells. Annu. Rev. Neurosci. 23, 285–314. 10.1146/annurev.neuro.23.1.285 10845066

[B16] Emmett S. D. Francis H. W. (2015). The socioeconomic impact of hearing loss in U.S. adults. Otol. Neurotol. 36, 545–550. 10.1097/MAO.0000000000000562 25158616 PMC4466103

[B17] Ernest S. Rauch G. J. Haffter P. Geisler R. Petit C. Nicolson T. (2000). Mariner is defective in myosin VIIA: a zebrafish model for human hereditary deafness. Hum. Mol. Genet. 9, 2189–2196. 10.1093/hmg/9.14.2189 10958658

[B18] Esterberg R. Coffin A. B. Ou H. Simon J. A. Raible D. W. Rubel E. W. (2013). Fish in a dish: drug discovery for hearing habilitation. Drug Discov. Today Dis. Models 10, e23–e29. 10.1016/j.ddmod.2012.02.001 24187569 PMC3811936

[B19] Fee W. E. (1980). Aminoglycoside ototoxicity in the human. Laryngoscope 90, 1–19. 10.1288/00005537-198010001-00001 7432055

[B20] Ferguson D. (2008). A study of clinical strains of *Pseudomonas aeruginosa* and the investigation of antibiotic resistance mechanisms in the multidrug resistant strain PA13 (thesis). Available online at: http://doras.dcu.ie/105/. Accessed September 2015.

[B21] Forge A. Li L. (2000). Apoptotic death of hair cells in mammalian vestibular sensory epithelia. Hear. Res. 139, 97–115. 10.1016/s0378-5955(99)00177-x 10601716

[B22] Forge A. Schacht J. (2000). Aminoglycoside antibiotics. Audiol. Neurootol. 5, 3–22. 10.1159/000013861 10686428

[B23] Furness D. N. (2015). Molecular basis of hair cell loss. Cell Tissue Res. 361, 387–399. 10.1007/s00441-015-2113-z 25676005

[B24] Gale J. E. Marcotti W. Kennedy H. J. Kros C. J. Richardson G. P. (2001). FM1–43 dye behaves as a permeant blocker of the hair-cell mechanotransducer channel. J. Neurosci. 21, 7013–7025. 11549711 10.1523/JNEUROSCI.21-18-07013.2001 PMC6762973

[B25] Harris J. Cheng A. Cunningham L. MacDonald G. Raible D. Rubel E. (2003). Neomycin-induced hair cell death and rapid regeneration in the lateral line of zebrafish (*Danio rerio*). J. Assoc. Res. Otolaryngol. 4, 219–234. 10.1007/s10162-002-3022-x 12943374 PMC3202713

[B26] Hashino E. Shero M. (1995). Endocytosis of aminoglycoside antibiotics in sensory hair cells. Brain Res. 704, 135–140. 10.1016/0006-8993(95)01198-6 8750975

[B27] He Y. Tang D. Cai C. Chai R. Li H. (2015). LSD1 is required for hair cell regeneration in zebrafish. Mol. Neurobiol. [Epub ahead of print]. 10.1007/s12035-015-9206-2 26008620

[B28] Hirose K. Westrum L. E. Cunningham D. E. Rubel E. W. (2004). Electron microscopy of degenerative changes in the chick basilar papilla after gentamicin exposure. J. Comp. Neurol. 470, 164–180. 10.1002/cne.11046 14750159

[B29] Hoekstra D. Janssen J. (1983). Non-visual feeding behavior of the mottled sculpin, *Cottus bairdi*, in Lake Michigan. Environ. Biol. Fish. 12, 111–117. 10.1007/bf00002763

[B30] Howard J. Roberts W. M. Hudspeth A. J. (1988). Mechanoelectrical transduction by hair cells. Annu. Rev. Biophys. Biophys. Chem. 17, 99–124. 10.1146/annurev.biophys.17.1.99 3293600

[B31] Huth M. E. Ricci A. J. Cheng A. G. (2011). Mechanisms of aminoglycoside ototoxicity and targets of hair cell protection. Int. J. Otolaryngol. 2011:937861. 10.1155/2011/937861 22121370 PMC3202092

[B32] Ji H. Li X. Zhang H. (2009). Natural products and drug discovery. EMBO Rep. 10, 194–200. 10.1038/embor.2009.12 19229284 PMC2658564

[B33] Jiang H. Sha S.-H. H. Forge A. Schacht J. (2006). Caspase-independent pathways of hair cell death induced by kanamycin *in vivo*. Cell Death Differ. 13, 20–30. 10.1038/sj.cdd.4401706 16021180 PMC1525047

[B34] Jørgensen F. Ohmori H. (1988). Amiloride blocks the mechano-electrical transduction channel of hair cells of the chick. J. Physiol. 403, 577–588. 10.1113/jphysiol.1988.sp017265 2473197 PMC1190729

[B35] Kazmierczak P. Sakaguchi H. Tokita J. Wilson-Kubalek E. M. Milligan R. A. Müller U.. (2007). Cadherin 23 and protocadherin 15 interact to form tip-link filaments in sensory hair cells. Nature 449, 87–91. 10.1038/nature06091 17805295

[B36] Kochkin S. (2007). The impact of untreated hearing loss on household income. Available online at: http://www.betterhearing.org/sites/default/files/hearingpedia-resources/M7_Hearing_aids_and_income_2006.pdf. Accessed on July 2015.

[B37] Küpeli E. Koşar M. Yeşilada E. Hüsnü K. Başer C. (2002). A comparative study on the anti-inflammatory, antinociceptive and antipyretic effects of isoquinoline alkaloids from the roots of Turkish *Berberis* species. Life Sci. 72, 645–657. 10.1016/s0024-3205(02)02200-2 12467905

[B38] Kuroda H. Nakazawa S. Katagiri K. Shiratori O. Kozuka M. Fujitani K.. (1976). Antitumor effect of bisbenzylisoquinoline alkaloids. Chem. Pharm. Bull. (Tokyo) 24, 2413–2420. 10.1248/cpb.24.2413 1017086

[B39] Lenz D. R. Avraham K. B. (2011). Hereditary hearing loss: from human mutation to mechanism. Hear. Res. 281, 3–10. 10.1016/j.heares.2011.05.021 21664957

[B40] Low A. M. Berdik M. Sormaz L. Gataiance S. (1996). Plant alkaloids, tetrandrine and hernandezine, inhibit calcium-depletion stimulated calcium entry in human and bovine endothelial cells. Life Sci. 58, 2327–2335. 10.1016/0024-3205(96)00233-0 8649222

[B41] Ma E. Y. Rubel E. W. Raible D. W. (2008). Notch signaling regulates the extent of hair cell regeneration in the zebrafish lateral line. J. Neurosci. 28, 2261–2273. 10.1523/JNEUROSCI.4372-07.2008 18305259 PMC6671837

[B42] Maeda R. Kindt K. S. Mo W. Morgan C. P. Erickson T. Zhao H.. (2014). Tip-link protein protocadherin 15 interacts with transmembrane channel-like proteins TMC1 and TMC2. Proc. Natl. Acad. Sci. U S A 111, 12907–12912. 10.1073/pnas.1402152111 25114259 PMC4156717

[B43] Marcotti W. van Netten S. M. Kros C. J. (2005). The aminoglycoside antibiotic dihydrostreptomycin rapidly enters mouse outer hair cells through the mechano-electrical transducer channels. J. Physiol. 567, 505–521. 10.1113/jphysiol.2005.085951 15994187 PMC1474200

[B44] Matsui J. I. Gale J. E. Warchol M. E. (2004). Critical signaling events during the aminoglycoside-induced death of sensory hair cells *in vitro*. J. Neurobiol. 61, 250–266. 10.1002/neu.20054 15389694

[B45] Matsui J. I. Ogilvie J. M. Warchol M. E. (2002). Inhibition of caspases prevents ototoxic and ongoing hair cell death. J. Neurosci. 22, 1218–1227. 11850449 10.1523/JNEUROSCI.22-04-01218.2002 PMC6757575

[B46] Metcalfe W. K. Kimmel C. B. Schabtach E. (1985). Anatomy of the posterior lateral line system in young larvae of the zebrafish. J. Comp. Neurol. 233, 377–389. 10.1002/cne.902330307 3980776

[B47] Meyers J. R. MacDonald R. B. Duggan A. Lenzi D. Standaert D. G. Corwin J. T.. (2003). Lighting up the senses: FM1-43 loading of sensory cells through nonselective ion channels. J. Neurosci. 23, 4054–4065. 12764092 10.1523/JNEUROSCI.23-10-04054.2003 PMC6741082

[B48] Mick P. Kawachi I. Lin F. R. (2014). The association between hearing loss and social isolation in older adults. Otolaryngol. Head Neck Surg. 150, 378–384. 10.1177/0194599813518021 24384545

[B49] Montgomery J. Hamilton A. (1997). Sensory contributions to nocturnal prey capture in the dwarf scorpion fish (*Scorpaena papillosus*). Mar. Freshwater. Behav. Physiol. 30, 209–223. 10.1080/10236249709379026

[B50] Murakami S. L. Cunningham L. L. Werner L. A. Bauer E. Pujol R. Raible D. W.. (2003). Developmental differences in susceptibility to neomycin-induced hair cell death in the lateral line neuromasts of zebrafish (*Danio rerio*). Hear. Res. 186, 47–56. 10.1016/s0378-5955(03)00259-4 14644458

[B51] Nada S. E. Tulsulkar J. Shah Z. A. (2014). Heme oxygenase 1-mediated neurogenesis is enhanced by *Ginkgo biloba* (EGb 761°ledR) after permanent ischemic stroke in mice. Mol. Neurobiol. 49, 945–956. 10.1007/s12035-013-8572-x 24154866 PMC3954901

[B52] Namdaran P. Reinhart K. E. Owens K. N. Raible D. W. Rubel E. W. (2012). Identification of modulators of hair cell regeneration in the zebrafish lateral line. J. Neurosci. 32, 3516–3528. 10.1523/JNEUROSCI.3905-11.2012 22399774 PMC3318954

[B53] Nicolson T. (2005). The genetics of hearing and balance in zebrafish. Annu. Rev. Genet. 39, 9–22. 10.1146/annurev.genet.39.073003.105049 16285850

[B54] Nicolson T. Rüsch A. Friedrich R. W. Granato M. Ruppersberg J. P. Nüsslein-Volhard C. (1998). Genetic analysis of vertebrate sensory hair cell mechanosensation: the zebrafish circler mutants. Neuron 20, 271–283. 10.1016/s0896-6273(00)80455-9 9491988

[B55] Ou H. C. Cunningham L. L. Francis S. P. Brandon C. S. Simon J. A. Raible D. W.. (2009). Identification of FDA-approved drugs and bioactives that protect hair cells in the zebrafish (*Danio rerio*) lateral line and mouse (*Mus musculus*) utricle. J. Assoc. Res. Otolaryngol. 10, 191–203. 10.1007/s10162-009-0158-y 19241104 PMC2674201

[B56] Ou H. C. Keating S. Wu P. Simon J. A. Raible D. W. Rubel E. W. (2012). Quinoline ring derivatives protect against aminoglycoside-induced hair cell death in the zebrafish lateral line. J. Assoc. Res. Otolaryngol. 13, 759–770. 10.1007/s10162-012-0353-0 23053627 PMC3505584

[B57] Ou H. C. Raible D. W. Rubel E. W. (2007). Cisplatin-induced hair cell loss in zebrafish (*Danio rerio*) lateral line. Hear. Res. 233, 46–53. 10.1016/j.heares.2007.07.003 17709218 PMC2080654

[B58] Owens K. N. Coffin A. B. Hong L. S. Bennett K. O. Rubel E. W. Raible D. W. (2009). Response of mechanosensory hair cells of the zebrafish lateral line to aminoglycosides reveals distinct cell death pathways. Hear. Res. 253, 32–41. 10.1016/j.heares.2009.03.001 19285126 PMC3167481

[B59] Owens K. N. Cunningham D. E. MacDonald G. Rubel E. W. Raible D. W. Pujol R. (2007). Ultrastructural analysis of aminoglycoside-induced hair cell death in the zebrafish lateral line reveals an early mitochondrial response. J. Comp. Neurol. 502, 522–543. 10.1002/cne.21345 17394157

[B60] Owens K. N. Santos F. Roberts B. Linbo T. Coffin A. B. Knisely A. J.. (2008). Identification of genetic and chemical modulators of zebrafish mechanosensory hair cell death. PLoS Genet. 4:e1000020. 10.1371/journal.pgen.1000020 18454195 PMC2265478

[B61] Pan B. Géléoc G. S. Asai Y. Horwitz G. C. Kurima K. Ishikawa K.. (2013). TMC1 and TMC2 are components of the mechanotransduction channel in hair cells of the mammalian inner ear. Neuron 79, 504–515. 10.1016/j.neuron.2013.06.019 23871232 PMC3827726

[B62] Partridge B. L. Pitcher T. J. (1980). The sensory basis of fish schools: relative roles of lateral line and vision. J. Comp. Physiol. 135, 315–325. 10.1007/bf00657647

[B63] Pelletier W. (1996). Alkaloids: Chemical and Biological Perspectives: Volume 11. Tarrytown, NY: Elsevier Science. Pergamon.

[B64] Raible D. W. Kruse G. J. (2000). Organization of the lateral line system in embryonic zebrafish. J. Comp. Neurol. 421, 189–198. 10.1002/(sici)1096-9861(20000529)421:2<189::aid-cne5>3.0.co;2-k 10813781

[B65] Ricci A. J. Fettiplace R. (1998). Calcium permeation of the turtle hair cell mechanotransducer channel and its relation to the composition of endolymph. J. Physiol. 506, 159–173. 10.1111/j.1469-7793.1998.159bx.x 9481679 PMC2230715

[B66] Rizzi M. D. Hirose K. (2007). Aminoglycoside ototoxicity. Curr. Opin. Otolaryngol. Head Neck Surg. 15, 352–357. 10.1097/MOO.0b013e3282ef772d 17823553

[B67] Russell I. J. Sellick P. M. (1976). Measurement of potassium and chloride ion concentrations in the cupulae of the lateral lines of *Xenopus laevis*. J. Physiol. 257, 245–255. 10.1113/jphysiol.1976.sp011366 948058 PMC1309354

[B68] Santos F. MacDonald G. Rubel E. W. Raible D. W. (2006). Lateral line hair cell maturation is a determinant of aminoglycoside susceptibility in zebrafish (*Danio rerio*). Hear. Res. 213, 25–33. 10.1016/j.heares.2005.12.009 16459035

[B69] Schacht J. Talaska A. Rybak L. (2012). Cisplatin and aminoglycoside antibiotics: hearing loss and its prevention. Anat. Rec. (Hoboken) 295, 1837–1850. 10.1002/ar.22578 23045231 PMC3596108

[B70] Schif P. L. (1991). Bisbenzylisoquinoline alkaloids. J. Nat. Prod. 54, 645–749. 10.1021/np50075a001 1955879

[B71] Self T. Mahony M. Fleming J. Walsh J. Brown S. D. Steel K. P. (1998). Shaker-1 mutations reveal roles for myosin VIIA in both development and function of cochlear hair cells. Development 125, 557–566. 9435277 10.1242/dev.125.4.557

[B72] Söllner C. Rauch G. J. Siemens J. Geisler R. Schuster S. C. Müller U.. (2004). Mutations in cadherin 23 affect tip links in zebrafish sensory hair cells. Nature 428, 955–959. 10.1038/nature02484 15057246

[B73] Steyger P. S. Peters S. L. Rehling J. Hordichok A. (2003). Uptake of gentamicin by bullfrog saccular hair cells *in vitro*. J. Assoc. Res. Otolaryngol. 4, 565–578. 10.1007/s10162-003-4002-5 14605921 PMC3202742

[B74] Suli A. Guler A. D. Raible D. W. Kimelman D. (2014). A targeted gene expression system using the trypotophan repressor in zebrafish shows no silencing in subsequent generations. Development 141, 1167–1174. 10.1242/dev.100057 24550120 PMC3929415

[B75] Thomas A. J. Hailey D. W. Stawicki T. M. Wu P. Coffin A. B. Rubel E. W.. (2013). Functional mechanotransduction is required for cisplatin-induced hair cell death in the zebrafish lateral line. J. Neurosci. 33, 4405–4414. 10.1523/JNEUROSCI.3940-12.2013 23467357 PMC3666553

[B76] Thomas A. J. Wu P. Raible D. W. Rubel E. W. Simon J. A. Ou H. C. (2015). Identification of small molecule inhibitors of cisplatin-induced hair cell death: results of a 10,000 compound screen in the zebrafish lateral line. Otol. Neurotol. 36, 519–525. 10.1097/MAO.0000000000000487 25687728 PMC4332566

[B77] Ton C. Parng C. (2005). The use of zebrafish for assessing ototoxic and otoprotective agents. Hear. Res. 208, 79–88. 10.1016/j.heares.2005.05.005 16014323

[B78] Trapani J. G. Nicolson T. (2010). Physiological recordings from zebrafish lateral-line hair cells and afferent neurons. Methods Cell Biol. 100, 219–231. 10.1016/b978-0-12-384892-5.00008-6 21111219

[B79] Uribe P. M. Mueller M. A. Gleichman J. S. Kramer M. D. Wang Q. Sibrian-Vazquez M.. (2013). Dimethyl sulfoxide (DMSO) exacerbates cisplatin-induced sensory hair cell death in zebrafish (*Danio rerio*). PLoS One 8:e55359. 10.1371/journal.pone.0055359 23383324 PMC3562182

[B80] Valli P. Zucca G. Casella C. (1977). The importance of potassium in the function of frog semicircular canals. Acta Otolaryngol. 84, 344–351. 10.3109/00016487709123976 303425

[B81] Van Netten S. M. (1997). Hair cell mechano-transduction: its influence on the gross mechanical characteristics of a hair cell sense organ. Biophys. Chem. 68, 43–52. 10.1016/s0301-4622(97)00006-9 9468609

[B82] Van Trump W. J. Coombs S. Duncan K. McHenry M. J. (2010). Gentamicin is ototoxic to all hair cells in the fish lateral line system. Hear. Res. 261, 42–50. 10.1016/j.heares.2010.01.001 20060460

[B83] Vázquez-Espinosa E. Girón R. M. Gómez-Punter R. M. García-Castillo E. Valenzuela C. Cisneros C.. (2015). Long-term safety and efficacy of tobramycin in the management of cystic fibrosis. Ther. Clin. Risk Manag. 11, 407–415. 10.2147/TCRM.s75208 25792839 PMC4362982

[B84] Vlasits A. L. Simon J. A. Raible D. W. Rubel E. W. Owens K. N. (2012). Screen of FDA-approved drug library reveals compounds that protect hair cells from aminoglycosides and cisplatin. Hear. Res. 294, 153–165. 10.1016/j.heares.2012.08.002 22967486 PMC3626493

[B85] Vu A. A. Nadaraja G. S. Huth M. E. Luk L. Kim J. Chai R.. (2013). Integrity and regeneration of mechanotransduction machinery regulate aminoglycoside entry and sensory cell death. PLoS One 8:e54794. 10.1371/journal.pone.0054794 23359017 PMC3554584

[B86] Wang Q. Steyger P. S. (2009). Trafficking of systemic fluorescent gentamicin into the cochlea and hair cells. J. Assoc. Res. Otolaryngol. 10, 205–219. 10.1007/s10162-009-0160-4 19255807 PMC2674203

[B87] Weber J.-F. Fournet A. (1989). Alkaloidal content of four *Berberis* species. Structure of berberilaurine, a new bisbenzyltetrahydroisoquinoline. J. Nat. Prod. 52, 81–84. 10.1021/np50061a010

[B88] Westerfield M. (2000). The Zebrafish Book: A Guide for the Laboratory Use of Zebrafish (Danio Rerio). Eugene: University of Oregon.

[B89] World Health Organization (2015). Deafness and hearing loss. Available online at: http://www.who.int/mediacentre/factsheets/fs300/en/ Accessed on July 2015.

[B90] Xiao T. Roeser T. Staub W. Baier H. (2005). A GFP-based genetic screen reveals mutations that disrupt the architecture of the zebrafish retinotectal projection. Development 132, 2955–2967. 10.1242/dev.01861 15930106

[B91] Xie J. Talaska A. E. Schacht J. (2011). New developments in aminoglycoside therapy and ototoxicity. Hear. Res. 281, 28–37. 10.1016/j.heares.2011.05.008 21640178 PMC3169717

[B92] Zarei A. Changizi-Ashtiyani S. Taheri S. Ramezani M. (2015). A quick overview on some aspects of endocrinological and therapeutic effects of *Berberis vulgaris* L. Avicenna J. Phytomed. 5, 485–497. 26693406 PMC4678494

